# Structurally diverse c-Myc inhibitors share a common mechanism of action involving ATP depletion

**DOI:** 10.18632/oncotarget.4327

**Published:** 2015-05-30

**Authors:** Huabo Wang, Lokendra Sharma, Jie Lu, Paul Finch, Steven Fletcher, Edward V. Prochownik

**Affiliations:** ^1^ Division of Hematology/Oncology, Children's Hospital of Pittsburgh of University of Pittsburgh Medical Center, Pittsburgh PA, USA; ^2^ Department of Pharmaceutical Sciences, The University of Maryland School of Pharmacy, Baltimore, MD, USA; ^3^ The Greenebaum Cancer Center, Baltimore MD, USA; ^4^ Department of Microbiology and Molecular Genetics, The University of Pittsburgh, Pittsburgh, PA, USA; ^5^ The University of Pittsburgh Cancer Institute, Pittsburgh, PA, USA

**Keywords:** 10058-F4, 10074-G5, JQ1, artemisinin, glycolysis

## Abstract

The c-Myc (Myc) oncoprotein is deregulated in a large proportion of diverse human cancers. Considerable effort has therefore been directed at identifying pharmacologic inhibitors as potential anti-neoplastic agents. Three such groups of small molecule inhibitors have been described. The first is comprised of so-called “direct” inhibitors, which perturb Myc's ability to form productive DNA-binding heterodimers in association with its partner, Max. The second group is comprised of indirect inhibitors, which largely function by targeting the BET-domain protein BRD4 to prevent the proper formation of transcriptional complexes that assemble in response to Myc-Max DNA binding. Thirdly, synthetic lethal inhibitors cause the selective apoptosis of Myc over-expressing either by promoting mitotic catastrophe or altering Myc protein stability. We report here a common mechanism by which all Myc inhibitors, irrespective of class, lead to eventual cellular demise. This involves the depletion of ATP stores due to mitochondrial dysfunction and the eventual down-regulation of Myc protein. The accompanying metabolic de-regulation causes neutral lipid accumulation, cell cycle arrest, and an attempt to rectify the ATP deficit by up-regulating AMP-activated protein kinase (AMPK). These responses are ultimately futile due to the lack of functional Myc to support the requisite anabolic response. Finally, the effects of Myc depletion on ATP levels, cell cycle arrest, differentiation and AMPK activation can be mimicked by pharmacologic inhibition of the mitochondrial electron transport chain without affecting Myc levels. Thus, all Myc inhibitors promote a global energy collapse that appears to underlie many of their phenotypic consequences.

## INTRODUCTION

c-Myc (Myc) is among the most frequently de-regulated oncoproteins encountered in human cancers and is a well-studied cause of numerous experimental cancers [1-3]. As a result, considerable effort has been directed at identifying small molecule inhibitors of Myc or selective downstream Myc target gene products that are necessary to confer or maintain the transformed phenotype [4-7]. The importance of this goal is underscored by the fact that Myc is required for the proliferation and/or survival of many cancers even when it is not obviously deregulated [8, 9]. Thus, unlike most targeted therapies, which are typically directed against specific tumor types harboring disease-specific mutant forms of driver oncoproteins [10, 11], Myc represents a more universal target. However, the fact that Myc is seldom mutated in human cancer, possesses no readily targetable enzymatic activities and is also expressed by normal proliferating cells raises considerable challenges to the eventual implementation of such specific therapies without engendering undue toxicities [5-7].

To date, three broad groups of small molecule Myc inhibitors have been identified which we have previously classified into “direct”, “indirect” and “synthetic lethal” categories [5]. Direct inhibitors are represented by molecules such as 10058-F4, 10074-G5, IIa6B17, JKY-2-169 and others which affect the interaction between Myc and its obligate bHLH-ZIP hetero-dimerization partner Max [12-17]. In so doing, these inhibitors either prevent heterodimer formation or alter its conformation in a manner that renders it incapable of binding its target DNA [12, 17]. In either case, they interfere with Myc's role in activating transcription by promoting acetylation-mediated chromatin relaxation and RNA Pol II-mediated promoter-proximal transcriptional pause release [18-20].

“Indirect” inhibitors, represented by the thieno-triazolo-1,4-diazepine JQ1 [21] do not affect Myc-Max heterodimerization. Rather, they act downstream of DNA binding by competitively inhibiting binding of the BET domain protein BRD4 to acetylated lysine residues of histone H3, thereby attenuating Myc-mediated transcriptional up-regulation [22-24]. The reduced density of histone H3 acetylation at intended Myc target genes also likely serves to reduce the efficiency with which promoter pause-release factors such as pTEFb and promoter clearance factors such as TFII-H are recruited to the Myc-Max transcriptional complex. Because the *MYCC* gene is heavily bound by BRD4 at a highly acetylated region approximately 2 kb upstream of the transcriptional start site, JQ1 treatment also inhibits Myc transcript and protein expression in some cell types [22, 23]. The combination of reduced BRD4 binding at both Myc target genes and the *MYCC* gene itself likely accounts for the high specificity and potency of this compound in some human cancers.

Lastly, synthetic lethal Myc inhibitors also act indirectly but differ from true indirect inhibitors in that they selectively promote tumor cell proliferative arrest and/or apoptosis only when Myc is clearly deregulated and over-expressed. Included among this group are inhibitors of GSK3β, which phosphorylates and de-stabilizes Myc via ubiquitin-mediated proteolysis [25]. The resultant pathological accumulation of Myc protein in the face of these compounds may trigger apoptosis. Other types of synthetic lethal inhibitors include compounds targeting CDK1 and Aurora B kinases, which are required for the proper assembly and function of the mitotic spindle [26, 27] and derivatives of the anti-malarial compound artemisinin, which presumably de-stabilize Myc by increasing rather than inhibiting GSK3β and promoting more efficient Myc protein degradation in tumors whose survival is highly Myc-dependent [28]. As a group, these synthetic lethal inhibitors seem to promote tumor cell demise either by altering the balance of Myc protein needed for tumor cell viability or by capitalizing upon Myc's tendency to promote aneuploidy [13, 29] by compromising the transformed cell's ability to faithfully partition its abnormal chromosome complement.

In the current work, we have tested representative compounds from each of these three groups of inhibitors and show that, despite their widely differing chemical structures and means of inhibiting Myc, they share a common core mechanism that involves the depletion of cellular ATP. Because Myc is needed to sustain glycolysis, mitochondrial biogenesis and oxidative phosphorylation (Oxphos) [30-32], the loss of its function upon inhibitor treatment leads to a rapid suppression of these energy-generating pathways and terminal differentiation when this course is an option or apoptotic demise when it is not. Myc inhibitor-treated cells respond to the loss of ATP by appropriately activating AMP-activated protein kinase (AMPK), a serine/threonine kinase that normally replenishes ATP by promoting glycolysis and Oxphos [33-35]. However, AMPK activation is ultimately futile due to the inability of the Myc inhibitor-treated cells to up-regulate these Myc-dependent processes. Collectively, these studies underscore the importance of Myc in maintaining the high anabolic demands of proliferating tumor cells. Thus, irrespective of their class, Myc inhibitors ultimately exert a common inhibitory effect on cancer cells by promoting an irreversible global energy collapse.

## RESULTS

### Disparate classes of Myc inhibitors promote HL60 cell cycle arrest and differentiation

For the studies reported here, we selected 9 direct, indirect and synthetic-lethal Myc inhibitors as representative of their class (Supplementary Figure 1). Within the first class were two previously well-characterized compounds, 10058-F4 and 10074-G5 [13, 18, 36, 37], along along with two more potent analogs of each: 12Rh and 28Rh for 10058-F4 and 3JC-91-2 and 3JC-91-7 for 10074-G5 [12, 15, 38]. Extensive analyses 10058-F4 and 10074-G5 have shown them to bind to geographically distinct regions of Myc's intrinsically disordered monomeric bHLH-ZIP dimerization domain where they promote a regional conformational distortion and prevent heterodimerization with Max, Myc's obligate bHLH-ZIP partner protein [36, 39-43]. An additional direct small molecule inhibitor with a mechanism of action distinct from that of 10058-F4 and 10074-G5 and their analogs was the recently described JKY-2-169, a proteomimetic, that was specifically designed to interact with Myc only in its α−helical conformation that it assumes upon dimerizing with Max [17, 44]. We have shown that JKY-2-169 promotes the loss of DNA binding by perturbing the conformation of Myc-Max heterodimers without causing their dissociation [17].

Representing the indirect class of inhibitors was JQ1, which binds to the acetyl lysine recognition domain of BRD4 [21]. DNA binding by Myc-Max heterodimers in the nucleus initiates chromatin remodeling by recruiting a multi-protein complex that includes histone acetylases such as GCN5 and TIP60 [23]. The subsequent recruitment of BRD4 to sites of dense histone H3 acetyl lysine content is necessary to abrogate transcriptional pausing and the promotion of uni-directional read-through by RNA Pol II.

Lastly, and representative of the synthetic lethal class of Myc inhibitors, was dihydro artemisinin (DHA), a common metabolite of the potent anti-malarial drug artemisinin and its analogs, which has been shown to selectively promote the proliferative arrest and apoptosis of Myc over-expressing cells [28, 45]. Although the precise means by which DHA promotes these phenotypes remains to be determined, it appears to destabilize Myc protein by promoting its ubiquitin-dependent proteasomal degradation [28, 45]. Because many tumors have been proposed to be dependent upon or “addicted” to high-level Myc expression [46, 47], its loss may selectively impair their survival.

**Figure 1:**
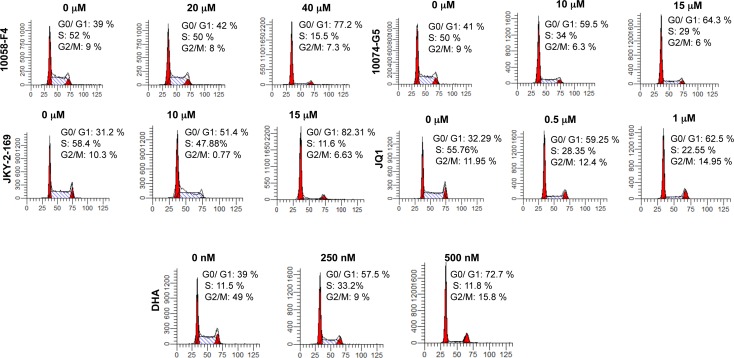
Myc inhibitors of different classes promote Go/G1 arrest HL60 cells in log-phase growth were plated into fresh medium at a concentration of ca. 10^5^ cells/ml and incubated for 24-48 hr in the presence of the indicated concentrations of each Myc inhibitor. The cells were then stained with propidium iodide and subjected to cell cycle analysis as previously described [83]. See Supplementary Figure. 2 for similar analyses with additional analogs.

Because Myc is necessary to drive the proliferation of virtually all transformed cells [8, 9, 46], we first examined the cell cycle effects on the above-described Myc inhibitors in HL60 promyelocytic leukemia cells, which over-express Myc due to gene amplification and which have been previously used to assess many Myc inhibitors [12, 15, 38, 48]. As seen in Figure 1 and Supplementary Figure 2, all the above Myc inhibitors induced a dose-dependent Go/G1 arrest which is a common response in many cell types depleted of Myc either pharmacologically or genetically [9, 19, 28]. In most cases, maximal arrest was observed within 24 hr of inhibitor addition with longer periods of exposure leading to apoptosis (Supplementary Figure 3).

**Figure 2:**
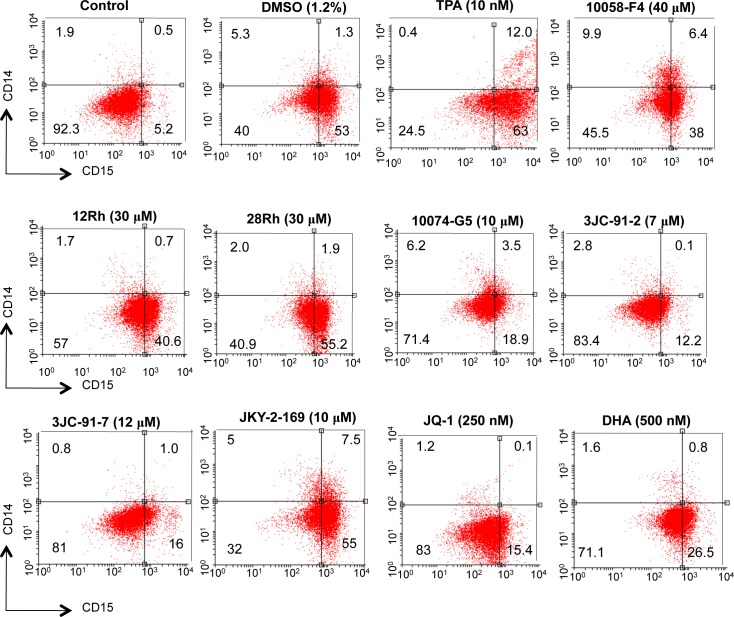
Myc inhibitors promote myeloid differentiation of HL60 cells HL60 cells in log-phase growth (ca. 10^5^ cells/ml) were incubated with the indicated concentrations of Myc inhibitors for 4-5 days at which point they were stained with mAbs directed against cell surface CD14 and CD15. Separate cultures were incubated with DMSO or 12-O-tetradecanoylphorbol-13-acetate (TPA), as controls for “pure” myeloid and monocyte/macrophage differentiation, respectively. Cell surface fluorescence was evaluated by two-color flow cytometry.

Inhibition of Myc in HL60 and other leukemias promotes differentiation. Moreover, chemically-induced differentiation by agents such as dimethyl sulfoxide is typically preceded by a rapid down-regulation of Myc [23, 28, 49-52]. Moreover, differentiation in these and many other cell types can be inhibited by the enforced over-expression of Myc or its v-Myc counterpart [52-56]. To determine whether the Go/G1 arrest observed with Myc inhibitor-treated cells was associated with a differentiated phenotype, HL60 cells were treated for 5 days with representative inhibitors from each of the groups and the surviving cells were then assessed for the expression of differentiation-specific cell surface markers using fluorescently-tagged mAbs that recognize the myeloid-specific antigen CD15 and the macrophage/monocyte antigen CD14 [57]. Control cells were either left untreated or were exposed to DMSO or the phorbol ester 12-O-tetradecanoylphorbol-13-acetate (TPA), which induce myeloid and macrophage/monocyte differentiation, respectively [58, 59]. As seen in Figure 2, DMSO up-regulated CD15 10-fold while only minimally increasing CD14 expression (approx. 2.5-fold). In contrast, treatment with TPA led to a concurrent up-regulation of both CD15 (12-fold) and CD14 (24-fold). By way of confirmation, Wright-Giemsa-stained samples of these same cells revealed the expected morphological changes associated with myeloid- and macrophage-specific differentiation (Supplementary Figure 4). All Myc inhibitors induced a predominantly myeloid phenotype, although with some variability. The modest degree of macrophage/monocyte differentiation observed by flow cytometry in response to JKY-2-169 (Figure 2) was not apparent from morphological assessment. Thus, consistent with previously identified roles for Myc, all three classes of Myc inhibitors promoted a primarily myeloid differentiation, as previously described for some of these agents [28, 60].

### Myc inhibitors promote neutral lipid accumulation and mitochondrial dysfunction

**Figure 3:**
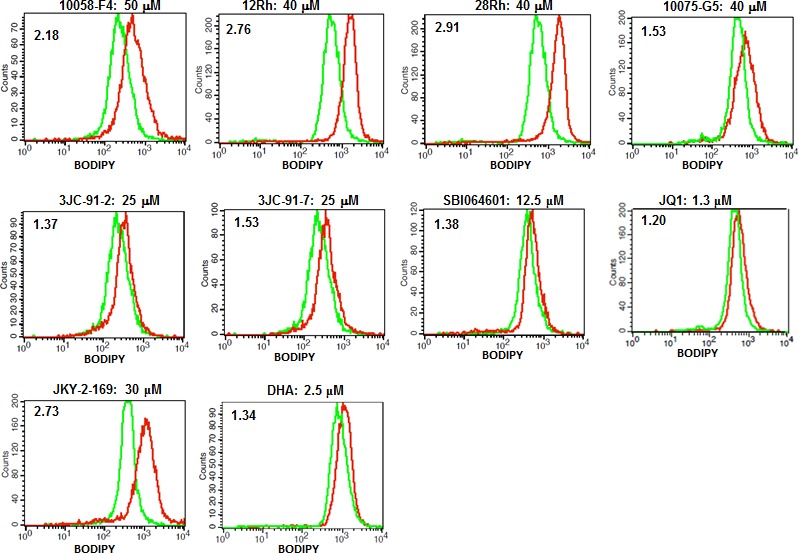
Myc inhibitors promote accumulation of neutral lipids H460 lung cancer cells were exposed to the indicated concentrations of Myc inhibitors for 3 days and then stained for neutral lipids using BODIPY-493/503. Ratios of the mean staining intensity of inhibitor-treated (red curves) to vehicle (DMSO)-treated (green curves) cells are indicated in the upper left of each histogram.

Previous studies have shown Myc to be necessary for maintaining and regulating cellular energy levels in the form of ATP [32, 61]. In Myc's absence, mitochondrial mass is significantly reduced and the remaining organelles become atrophic, decrease their rates of Oxphos, display abnormalities in ETC structure and function and sharply curtail their production of ATP [31, 32]. For example, basal ATP levels in *myc−/−* fibroblasts are only about 20% those of their *myc+/+* counterparts despite the former cells having slower growth rates and reduced ATP consumption [32]. Because Myc also regulates glycolysis [30, 32, 62, 63], the profound energy deficit cannot be reversed simply by up-regulating this pathway. The increased cytoplasmic accumulation of neutral lipids that has been described in Myc- and N-Myc depleted cells [61, 64] likely arises as the result of an increased utilization of fatty acids for β−oxidation as a way of compensating for the profound mitochondrial dysfunction [61]. However, because their rate of uptake exceeds their rate of metabolism, the excess fatty acids are stored as neutral lipid. We therefore hypothesized that all Myc inhibitors might ultimately converge on a common pathway leading to mitochondrial dysfunction and fatty acid accumulation. To test this, H460 lung cancer cells, which are quite sensitive to Myc levels [9], were exposed to representative Myc inhibitors for two days and then stained with the fluorescent, cell-permeable and neutral lipid-specific dye BODIPY-493/503. Flow cytometry-based quantification of BODIPY-493/503 uptake indicated that all Myc inhibitors caused significant increases in the accumulation of neutral lipids although the magnitude of the change in response to JQ1 and DHA was low (Figure 3).

**Figure 4:**
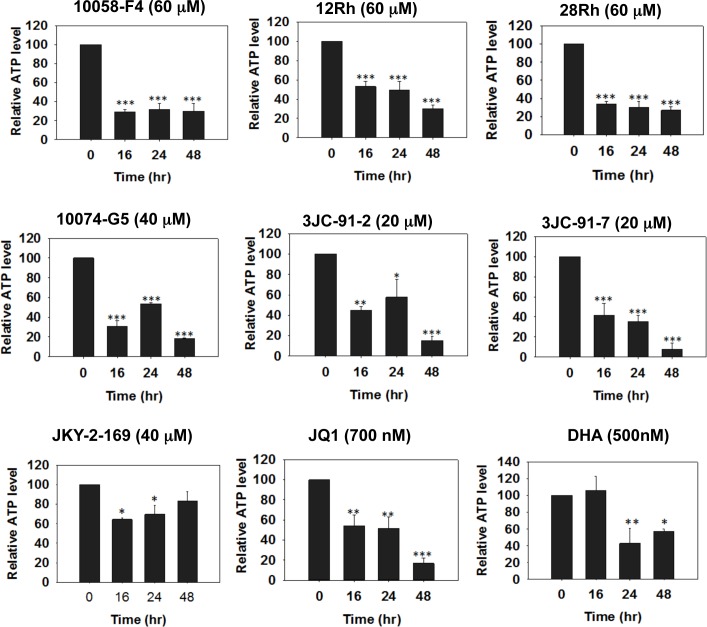
Myc inhibitors deplete cellular ATP Myc inhibitors were added to HL60 cells for the indicated periods of time at which point they were harvested and assayed for total ATP levels. Mean values of quadruplicate determinations +/− 1 SE are shown with the total ATP levels in untreated cells arbitrarily adjusted to 100%. All p values are described relative to untreated cells. *: p<0.05; **: p<0.01: *** : p<0.005. See Supplementary Figures 5 and 6 for the results of identical studies performed in H460 and CaLu1 lung cancer cells, respectively.

To further explore the consequences of Myc depletion on metabolism, we measured ATP levels in HL60, H460 and CaLu1 lung cancer cells following exposure to Myc inhibitors. As seen in Figure 4 for HL60 cells and Supplementary Figures 5 and 6 for H460 and CaLu1 cells, time-dependent decreases in ATP content were observed as early as 16 hr after the addition of most inhibitors and reached their nadir by 24-48 hr. Interestingly, although both JQ1 and DHA were able to reduce ATP levels in HL60 and CaLu1 cells (Supplementary Figures 5 and 6), they did so only minimally in H460 cells, thus suggesting an explanation for the inability of these inhibitors to promote anything more than only modest amounts of neutral lipid accumulation (Figure 3). Indeed, when BODIPY-493/503 staining was repeated on HL60 cells exposed to these inhibitors, a much more robust accumulation of neutral lipid was observed that correlated well with the correspondingly lower ATP levels (Supplementary Figure 7). These findings are thus consistent with the idea that ATP depletion, initiated by Myc inhibitor-mediated mitochondrial dysfunction is ultimately responsible for the accumulation of neutral lipids [61, 64].

**Figure 5:**
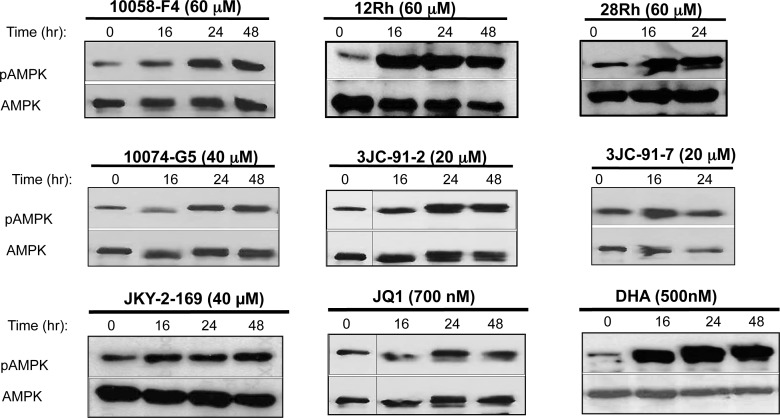
Myc inhibitors activate AMPK Myc inhibitors were added to log-phase HL60 cells for the indicated periods of time. The cells were then harvested and immuno-blotted for total AMPK or its activated, Thr_172_-phosphorylated form (pAMPK). See Supplementary Figures 8 and 9 for identical studies performed in H460 and CaLu1 cells.

One of the general cellular consequences of an energy-depleted state, irrespective of its cause, is an attempt to remedy the deficit by replenishing ATP. The means by which this is accomplished are intimately connected and largely mediated by the phosphorylation-dependent activation of AMPK [33, 35]. The pleiotropic consequences of this up-regulation include a marked reduction in energy-consuming process such as macromolecular bio-synthesis and cell proliferation and a stimulation of energy-producing processes such as glycolysis and Oxphos [33, 34]. To determine whether Myc inhibitors affected this pathway, we assessed total and phospho-Thr_172_-AMPK (pAMPK) in all three cell types following exposure to members of each of the three classes of Myc inhibitors. As seen in Figure 5 and Supplementary Figures 8 and 9, marked phosphorylation-dependent AMPK activation was observed in each case that largely paralleled the previously documented changes in ATP levels. That AMPK was activated in H460 cells in response to JQ1 and DHA, despite these compounds having little effect on cellular ATP or neutral lipid levels (Figure 3 and Supplementary Figure 5), suggested that even minor or transient declines in energy levels or perturbed mitochondrial function might be sufficient to trigger a response or that phosphorylation of AMPK was being regulated by factors other than the ATP:AMP ratio such as ROS that can arise as a result of mitochondrial dysfunction [65, 66]. Taken together, these results indicate that, while AMPK is, to a large degree, appropriately activated in response to Myc inhibitor-mediated ATP depletion, it is in most cases unable to restore a normal energy balance.

### Myc inhibitors promote Myc protein disappearance

**Figure 6:**
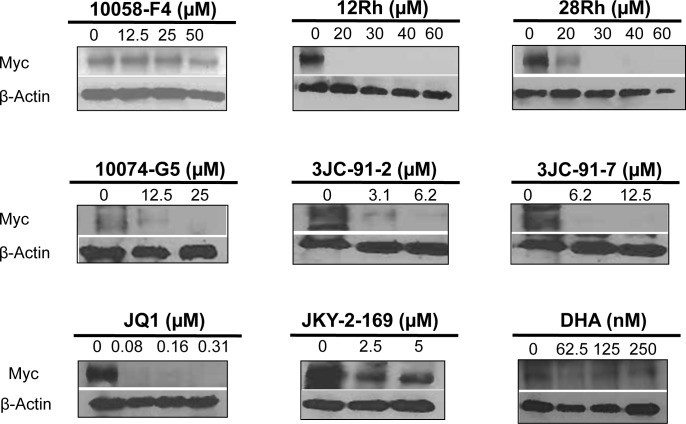
Myc protein immunoblots HL60 cells were exposed for 48 hr to the indicated concen-trations of Myc inhibitors. Cells were then assessed for Myc protein by standard immuno-blotting.

JQ1 has been reported to negatively regulate Myc expression by virtue of the fact that, in at least some cell types, the *MYCC* gene's promoter is a transcriptionally important site of BRD4 binding to acetylated histones [22]. In addition, DHA may regulate Myc protein stability by altering its proteasomal degradation [28]. We and others have also noticed that cells exposed to other direct Myc inhibitors for periods of time longer than are necessary to inhibit Myc activity also demonstrate lowered Myc protein levels [67, 68 and unpublished observations]. In order to determine whether mitochondrial dysfunction, ATP depletion and other phenotypes were associated with reduced Myc expression, we assessed Myc protein levels by standard immunoblotting at various times after exposure to the inhibitors. As shown in Figure 6 for HL60 cells and in Supplementary Figures 10 and 11 for H460 and CaLu1 lung cancer cells, each of the cell lines significantly down-regulation of Myc protein within 24-48 hr of the addition of most inhibitors. A notable exception was seen with H460 cells, which did not significantly down regulated Myc levels in response to JQ1 or DHA, thus likely explaining their unchanged ATP levels or BODIPY-493/503 staining (Figure 3 and Supplementary Figure 5). In the case of DHA, an eventual reduction in Myc protein was only seen at concentrations ~30-fold higher than were needed to suppress Myc protein in HL60 cells.

### ATP depletion alone mimics Myc inhibitor treatment

**Figure 7:**
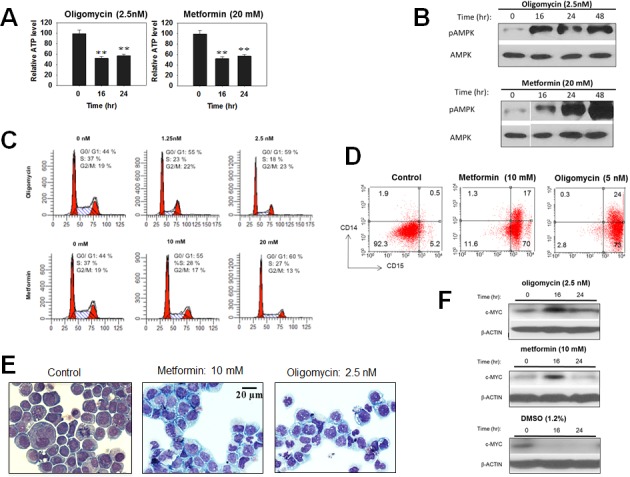
Disruption of the mitochondrial ETC mimics the effects of Myc inhibitors HL60 cells were exposed to the indicated concentrations of Metformin or Oligomycin for 48 hr. (**A**). ATP content. (**B**). Total and pAMPK immunoblots. (**C**). Cell cycle analyses. (**D**). Cell surface expression of CD14 and CD15. (**E**). Morphological appearance. (**F**). Myc protein immunoblots.

The foregoing findings suggested that a common mechanism to explain the effects of structurally diverse Myc inhibitors on cancer cell survival and/or differentiation might be their ability to deplete cellular ATP pools as a consequence of their dual effects on Myc protein abundance and function and, ultimately, on glycolysis and Oxphos. To test this directly we depleted ATP in HL60 cells with two structurally unrelated inhibitors of the mitochondrial electron transport chain (ETC), namely metformin, which inhibits Complex I [69-71] and oligomycin A, which inhibits Complex V (ATP synthase). Although the degree of ATP depletion achieved was somewhat less than that obtained with most Myc inhibitors (Figure 7A), robust AMPK activation with still observed and was associated with cell cycle arrest (Figures 7B and 7C). Concurrently, HL60 cells acquired a highly differentiated phenotype in which both CD14 and CD15 were even more highly expressed than in cells treated with Myc inhibitors, DMSO and TPA (Compare Figure 7D and Figure 2). Morphological changes were also observed and were more in keeping with were more in keeping with the more myelomonocytic form of differentiation induced by TPA (Figure 2) (Figure 7E). Finally, immunoblots for total Myc protein showed that unlike DMSO, whose induction of HL60 differentiation was associated with a nearly complete disappearance of Myc protein as previously described [72-75], neither metformin nor oligomycin caused such a pronounced decline (Figure 7F). From these studies, we conclude that ATP depletion alone is sufficient to promote many of the same phenotypes that are associated with Myc depletion.

## DISCUSSION

Pharmacologic inhibition of Myc has been demonstrated by us and others to occur through several mechanisms that include the prevention of Myc-Max association, the distortion of the Myc-Max heterodimer into a non-DNA binding complex, the interference of Myc-Max transcriptional regulation by inhibiting BRD4 and the enhanced proteolysis of the Myc protein itself [12-17, 23, 26-28, 38]. While these distinctions may be useful for heuristic purposes, they are undoubtedly somewhat over-simplified and their actual mechanisms are likely more heterogeneous and overlapping. For example, in addition to preventing BRD4 binding to acetylated histones on Myc target genes, JQ1 also induces Myc protein loss by inhibiting transcription of the *MYCC* gene whose own promoter also binds BRD4 at a densely acetylated site [22]. Indeed, the sensitivity of some multiple myelomas to JQ1 arises in part from a similar loss of Myc expression in cases where translocated *MYCC* coding sequences are juxtaposed to the IgH enhancer, which also binds BRD4 [23]. At this level then, JQ1 functionally resembles certain synthetic lethal inhibitors such as DHA, which destabilize Myc post-translationally by increasing its susceptibility to ubiquitin-mediated proteasomal degradation [28]. The transcriptional down-regulation of Myc that normally accompanies cellular quiescence may be an additional common feature of all inhibitors.

**Figure 8:**
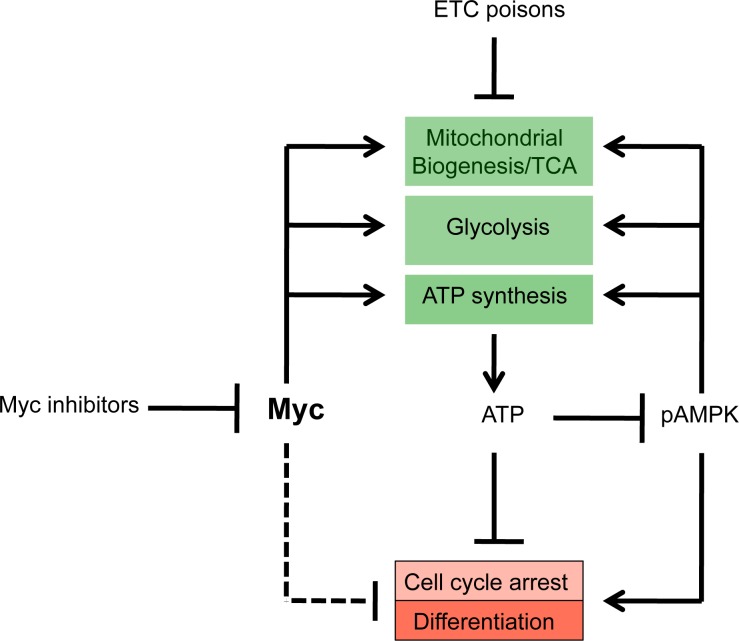
Common mechanism of Myc inhibitor action By virtue of their ability to prevent DNA binding by Myc-Max heterodimers and to promote Myc protein loss, Myc inhibitors down-regulate glycolysis and Oxphos [32, 61]. The ensuing depletion of cellular ATP pools leads to the activation of AMPK, which attempts to replenish ATP levels by down-regulating ATP-consuming processes such as proliferation and up-regulating ATP-generating processes such as glycolysis and OxPhos. However, because Myc is needed in concert to facilitate these functions, AMPK's effect is ultimately abortive and the cell remains chronically ATP-deprived. The lack of functional Myc also likely affects proliferation and differentiation independent of ATP levels as a result of failure to properly regulate genes encoding proteins necessary for cell cycle progression such as certain cyclin-dependent kinases and their inhibitors (dotted line) [82].

Irrespective of the means by which the different classes of Myc inhibitors limit the oncoprotein's function, it is clear from the work presented here that they ultimately converge on a single overriding mechanism that involves the depletion of cellular energy stores. According to this model (Figure 8), Myc over-expression by tumor cells is necessary to maximize glycolysis and Oxphos in order to support the high level of ATP consumption and biomass accumulation demanded by rapid, proliferation-associated anabolism [31, 32]. This coincides with significant increases in mitochondrial mass (and Oxphos) and explains how, relative to normal cells, those with de-regulated Myc can increase ATP turnover without affecting basal levels [32]. Conversely, Myc's absence leads to mitochondrial atrophy, ATP depletion and neutral lipid accumulation [31, 32, 64]. This latter phenotype has recently been shown to be due to an increase in fatty acid uptake and β−oxidation in an attempt to compensate for the impaired mitochondrial utilization of glucose and glutamine as energy-generating substrates [61]. An imbalance between fatty acid uptake and metabolism leads to the former outpacing the latter with the excess intracellular fatty acid being directed into neutral lipid stores. Even in the face of increased β−oxidation, the ATP deficit cannot be corrected due to the profound mitochondrial dysfunction. The accumulation of lipid droplets is likely further enhanced as a result of the reduced need to utilize their stored lipids and sterols for *de novo* membrane biogenesis following Myc depletion and the onset of proliferative arrest (Figure 1 and Supplementary Figure 2) [76-78].

The responses to ATP depletion that ensue as a result of Myc inhibitor exposure include Go/G1 arrest, which reduces anabolic demand. However, in appropriate cell types such as HL60 promyelocytes, terminal differentiation also occurs (Figure 2 and Supplementary Figure 4), thus ensuring that cell cycle arrest remains permanent. AMPK is also activated (Figure 5 and Supplementary Figs. 8 and 9), which, along with Myc inhibition, likely contributes further to cell cycle arrest [34]. In Myc-replete cells, the normal response to AMPK activation includes increases in glycolysis, mitochondrial biogenesis and Oxphos [33]. However, because these processes are highly Myc-dependent [31, 32, 61], they are impaired if the oncoprotein's function is disabled either by genetic or pharmacologic intervention. As a consequence, ATP levels are unable to normalize, thus likely explaining the eventual onset of apoptosis. Further supporting this idea is the finding that, in *myc−/−* fibroblasts, AMPK is chronically activated, ATP levels remain at 20% of normal and the cells show reductions in size, protein content and proliferation [32, 61].

That ATP depletion alone is sufficient to recapitulate most of the phenotypes elicited with Myc inhibitors (Figure 7) further supports the idea that these latter compounds ultimately serve to inhibit tumor growth by depleting cellular energy stores. However, Myc is well-known to control many genes involved in cell cycle progression and survival that operate through pathways quite distinct from those controlling metabolism [79-82]. Thus, Myc's effect on ATP levels likely provides only a partial explanation of how it regulates overall tumor cell fitness (Figure 8).

Collectively, our findings provide an empirical foundation upon which to suggest that maximal benefit from the clinical implementation of Myc inhibitors, regardless of their class, may derive from their combined use with agents that act to further compromise cellular energy supplies.

## MATERIALS AND METHODS

### Cell lines

HL60 human promyelocytic leukemia cells, human H460 large cell undifferentiated lung cancer cells and human CaLu1 non-small cell lung cancer cells were cultured as previously described [12, 38]. Briefly, HL60 cells were maintained in RPMI-1640 medium and H460 and CaLu1 cells were maintained in Dulbecco's modified Eagle's minimal essential medium (D-MEM). Both sets of media were supplemented with 10% heat-inactivated fetal bovine serum (FBS), 100 units/ml Penicillin G and 100 μg/ml streptomycin. All reagents were obtained from Atlanta Biologicals (Flowery Branch, GA).

### Cell cycle analysis

Sub-confluent cultures of cells at >90% viability were plated into fresh medium at densities that were typically about 10% of those attained at the point of density arrest. The next day, Myc inhibitors at the stated concentrations were added for 24-48 hr. The cells were then harvested, washed twice in PBS and stained with propidium iodide as previously described [29, 83]. Cell cycle analyses were performed on a BD FACSCalibur™ flow cytometer (Becton Dickinson, Inc. Franklin Lakes, NJ) and analyzed using ModFit LT 3.3 (Verity Software House, Topsham, ME).

### Myc inhibitors

10058-F4, 10074-G5, JQ1 and dihydroartemisinin (DHA) were purchased from Sigma-Aldrich (St. Louis, MO). The 10058-F4 analogs 12Rh and 28Rh, the 10074-G5 analogs 3JC-91-2 and 3JC91-7 and the Myc proteomimetic compound JKY-2-169 [17] were all synthesized and purified as previously described [12, 38].

### HL60 differentiation

HL60 cells were plated into fresh medium at approximately 10^5^ cells/ml and allowed to achieve log-phase growth overnight. They were then exposed to the indicated concentrations of Myc inhibitors for 4 days. Cell surface expression of CD14 and CD15 were then quantified using monoclonal antibodies against CD15 (clone HI98 Cat. #560997) and CD14 (clone M5E2 Cat. # 561707) (both from Becton-Dickinson) according to the directions of the supplier. Two color flow cytometry was performed on BD FACSCalibur™ flow cytometer and analyzed using CellQuest Pro software (BD Biosciences) and Flowing Software 2.5.1 (www.flowingsoftware.com).

### Neutral lipid measurements

Cells were seeded into 6 well plastic tissue culture plates (2 × 10^5^ cells/well and allowed to attach overnight. Fresh medium containing the indicated concentration of Myc inhibitor was then added and the incubation was continued for an additional 2-3 days. Cells were then trypsinized, washed in PBS and then stained in suspension with BODIPY-493/503 (Life Technologies, Carlsbad, CA) (2 μg/ml for 30 min). BODIPY-493/503 uptake was then assessed by flow cytometry using Cell Quest Pro software.

### ATP assays

Cells in 6-well plates were treated with Myc inhibitors for the indicated periods of time. The cells were then counted and lysed in 96 well plates. ATP determinations were performed using the ATP Lite ATP detection system (Perkin-Elmer, Inc. Downers Grove, IL) according to the directions of the supplier. Each point was assayed in quadruplicate and mean values +/− 1 standard error determined. ATP luminescence was normalized to the total number of cells used per assay and presented as a relative value compared with non-treated control. Statistical analyses were performed using GraphPad Prism 5 Software (GraphPad Software, Inc. La Jolla, CA) and p values were determined using Dunnett's Multiple Comparison Test.

### Western blotting

Cells were exposed to Myc inhibitors for the indicated periods of time and then harvested and lysed in RIPA buffer containing protease and phosphatase inhibitors. Equivalent amounts of protein were subjected to SDS-PAGE and immunoblotting according to previously-described procedures [12, 13]. Antibodies used included mouse monoclonal antibodies (mAbs) against AMPK (1:1000 dilution: catalog no. 2532, Cell Signaling Technology, Beverly, MA) and Myc (1:500 dilution: Clone 9E10, Santa Cruz Biotechnology, Inc. Santa Cruz, CA) a rabbit polyclonal antibody against AMPK phospho-Thr_172_ (1:1000 dilution, catalog no. 2535, Cell Signaling Technologies). As a control for protein loading, blots were also probed with a mouse mAb against β−actin (1:10,000 dilution: cat. no.3700S Cell Signaling Technology). Immunoblots were developed using an enhanced chemiluminescence reagent according to the directions of the supplier (SuperSignal West Femto Maxmum Sensitivity Substrate, Thermo Fisher Scientific, Inc. Waltham, MA).

## SUPPLEMENTARY MATERIAL FIGURES



## References

[1] Nesbit CE, Tersak JM, Prochownik EV (1999). MYC oncogenes and human neoplastic disease. Oncogene.

[2] Morton JP, Sansom OJ (2013). MYC-y mice: from tumour initiation to therapeutic targeting of endogenous MYC. Molecular oncology.

[3] Meyer N, Penn LZ (2008). Reflecting on 25 years with MYC. Nature reviews Cancer.

[4] Berg T (2011). Small-molecule modulators of c-Myc/Max and Max/Max interactions. Current topics in microbiology and immunology.

[5] Fletcher S, Prochownik EV (2014). Small-molecule inhibitors of the Myc oncoprotein. Biochimica et biophysica acta.

[6] Prochownik EV, Vogt PK (2010). Therapeutic Targeting of Myc. Genes & cancer.

[7] Prochownik EV (2004). c-Myc as a therapeutic target in cancer. Expert review of anticancer therapy.

[8] Soucek L, Whitfield J, Martins CP, Finch AJ, Murphy DJ, Sodir NM, Karnezis AN, Swigart LB, Nasi S, Evan GI (2008). Modelling Myc inhibition as a cancer therapy. Nature.

[9] Wang H, Mannava S, Grachtchouk V, Zhuang D, Soengas MS, Gudkov AV, Prochownik EV, Nikiforov MA (2008). c-Myc depletion inhibits proliferation of human tumor cells at various stages of the cell cycle. Oncogene.

[10] Evans MS, Madhunapantula SV, Robertson GP, Drabick JJ (2013). Current and future trials of targeted therapies in cutaneous melanoma. Advances in experimental medicine and biology.

[11] Bisen A, Claxton DF (2013). Tyrosine kinase targeted treatment of chronic myelogenous leukemia and other myeloproliferative neoplasms. Advances in experimental medicine and biology.

[12] Wang H, Hammoudeh DI, Follis AV, Reese BE, Lazo JS, Metallo SJ, Prochownik EV (2007). Improved low molecular weight Myc-Max inhibitors. Molecular cancer therapeutics.

[13] Yin X, Giap C, Lazo JS, Prochownik EV (2003). Low molecular weight inhibitors of Myc-Max interaction and function. Oncogene.

[14] Kiessling A, Wiesinger R, Sperl B, Berg T (2007). Selective inhibition of c-Myc/Max dimerization by a pyrazolo[1,5-a]pyrimidine. ChemMedChem.

[15] Yap JL, Wang H, Hu A, Chauhan J, Jung KY, Gharavi RB, Prochownik EV, Fletcher S (2013). Pharmacophore identification of c-Myc inhibitor 10074-G5. Bioorganic & medicinal chemistry letters.

[16] Berg T, Cohen SB, Desharnais J, Sonderegger C, Maslyar DJ, Goldberg J, Boger DL, Vogt PK (2002). Small-molecule antagonists of Myc/Max dimerization inhibit Myc-induced transformation of chicken embryo fibroblasts. Proceedings of the National Academy of Sciences of the United States of America.

[17] Jung KY, Wang H, Teriete P, Yap JL, Chen L, Lanning ME, Hu A, Lambert LJ, Holien T, Sudan A, Cosford ND, Prochownik EV, Fletcher S (2015). Perturbation of the c-Myc-Max Protein-Protein Interaction via Synthetic alpha-Helix Mimetics. Journal of medicinal chemistry.

[18] Rahl PB, Lin CY, Seila AC, Flynn RA, McCuine S, Burge CB, Sharp PA, Young RA (2010). c-Myc regulates transcriptional pause release. Cell.

[19] Lin CY, Loven J, Rahl PB, Paranal RM, Burge CB, Bradner JE, Lee TI, Young RA (2012). Transcriptional amplification in tumor cells with elevated c-Myc. Cell.

[20] Nie Z, Hu G, Wei G, Cui K, Yamane A, Resch W, Wang R, Green DR, Tessarollo L, Casellas R, Zhao K, Levens D (2012). c-Myc is a universal amplifier of expressed genes in lymphocytes and embryonic stem cells. Cell.

[21] Filippakopoulos P, Qi J, Picaud S, Shen Y, Smith WB, Fedorov O, Morse EM, Keates T, Hickman TT, Felletar I, Philpott M, Munro S, McKeown MR, Wang Y, Christie AL, West N (2010). Selective inhibition of BET bromodomains. Nature.

[22] Zuber J, Shi J, Wang E, Rappaport AR, Herrmann H, Sison EA, Magoon D, Qi J, Blatt K, Wunderlich M, Taylor MJ, Johns C, Chicas A, Mulloy JC, Kogan SC, Brown P (2011). RNAi screen identifies Brd4 as a therapeutic target in acute myeloid leukaemia. Nature.

[23] Delmore JE, Issa GC, Lemieux ME, Rahl PB, Shi J, Jacobs HM, Kastritis E, Gilpatrick T, Paranal RM, Qi J, Chesi M, Schinzel AC, McKeown MR, Heffernan TP, Vakoc CR, Bergsagel PL (2011). BET bromodomain inhibition as a therapeutic strategy to target c-Myc. Cell.

[24] Mertz JA, Conery AR, Bryant BM, Sandy P, Balasubramanian S, Mele DA, Bergeron L, Sims RJ (2011). Targeting MYC dependence in cancer by inhibiting BET bromodomains. Proceedings of the National Academy of Sciences of the United States of America.

[25] Rottmann S, Wang Y, Nasoff M, Deveraux QL, Quon KC (2005). A TRAIL receptor-dependent synthetic lethal relationship between MYC activation and GSK3beta/FBW7 loss of function. Proceedings of the National Academy of Sciences of the United States of America.

[26] Goga A, Yang D, Tward AD, Morgan DO, Bishop JM (2007). Inhibition of CDK1 as a potential therapy for tumors over-expressing MYC. Nature medicine.

[27] Yang D, Liu H, Goga A, Kim S, Yuneva M, Bishop JM (2010). Therapeutic potential of a synthetic lethal interaction between the MYC proto-oncogene and inhibition of aurora-B kinase. Proceedings of the National Academy of Sciences of the United States of America.

[28] Lu JJ, Meng LH, Shankavaram UT, Zhu CH, Tong LJ, Chen G, Lin LP, Weinstein JN, Ding J (2010). Dihydroartemisinin accelerates c-MYC oncoprotein degradation and induces apoptosis in c-MYC-overexpressing tumor cells. Biochemical pharmacology.

[29] Yin XY, Grove L, Datta NS, Katula K, Long MW, Prochownik EV (2001). Inverse regulation of cyclin B1 by c-Myc and p53 and induction of tetraploidy by cyclin B1 overexpression. Cancer research.

[30] Dang CV (2011). Therapeutic targeting of Myc-reprogrammed cancer cell metabolism. Cold Spring Harbor symposia on quantitative biology.

[31] Li F, Wang Y, Zeller KI, Potter JJ, Wonsey DR, O'Donnell KA, Kim JW, Yustein JT, Lee LA, Dang CV (2005). Myc stimulates nuclearly encoded mitochondrial genes and mitochondrial biogenesis. Molecular and cellular biology.

[32] Graves JA, Wang Y, Sims-Lucas S, Cherok E, Rothermund K, Branca MF, Elster J, Beer-Stolz D, Van Houten B, Vockley J, Prochownik EV (2012). Mitochondrial structure, function and dynamics are temporally controlled by c-Myc. PloS one.

[33] Hardie DG, Ross FA, Hawley SA (2012). AMPK: a nutrient and energy sensor that maintains energy homeostasis. Nature reviews Molecular cell biology.

[34] Hardie DG, Ross FA, Hawley SA (2012). AMP-activated protein kinase: a target for drugs both ancient and modern. Chemistry & biology.

[35] Yuan HX, Xiong Y, Guan KL (2013). Nutrient sensing, metabolism, and cell growth control. Molecular cell.

[36] Michel J, Cuchillo R (2012). The impact of small molecule binding on the energy landscape of the intrinsically disordered protein C-myc. PloS one.

[37] Zhang P, Metukuri MR, Bindom SM, Prochownik EV, O'Doherty RM, Scott DK (2010). c-Myc is required for the CHREBP-dependent activation of glucose-responsive genes. Molecular endocrinology.

[38] Wang H, Chauhan J, Hu A, Pendleton K, Yap JL, Sabato PE, Jones JW, Perri M, Yu J, Cione E, Kane MA, Fletcher S, Prochownik EV (2013). Disruption of Myc-Max heterodimerization with improved cell-penetrating analogs of the small molecule 10074-G5. Oncotarget.

[39] Cole MD, Cowling VH (2008). Transcription-independent functions of MYC: regulation of translation and DNA replication. Nature reviews Molecular cell biology.

[40] Nilsson JA, Cleveland JL (2003). Myc pathways provoking cell suicide and cancer. Oncogene.

[41] Follis AV, Hammoudeh DI, Wang H, Prochownik EV, Metallo SJ (2008). Structural rationale for the coupled binding and unfolding of the c-Myc oncoprotein by small molecules. Chemistry & biology.

[42] Follis AV, Hammoudeh DI, Daab AT, Metallo SJ (2009). Small-molecule perturbation of competing interactions between c-Myc and Max. Bioorganic & medicinal chemistry letters.

[43] Hammoudeh DI, Follis AV, Prochownik EV, Metallo SJ (2009). Multiple independent binding sites for small-molecule inhibitors on the oncoprotein c-Myc. Journal of the American Chemical Society.

[44] Nair SK, Burley SK (2003). X-ray structures of Myc-Max and Mad-Max recognizing DNA. Molecular bases of regulation by proto-oncogenic transcription factors. Cell.

[45] Morrissey C, Gallis B, Solazzi JW, Kim BJ, Gulati R, Vakar-Lopez F, Goodlett DR, Vessella RL, Sasaki T (2010). Effect of artemisinin derivatives on apoptosis and cell cycle in prostate cancer cells. Anti-cancer drugs.

[46] Arvanitis C, Felsher DW (2006). Conditional transgenic models define how MYC initiates and maintains tumorigenesis. Seminars in cancer biology.

[47] Pelengaris S, Khan M (2003). The c-MYC oncoprotein as a treatment target in cancer and other disorders of cell growth. Expert opinion on therapeutic targets.

[48] Mustata G, Follis AV, Hammoudeh DI, Metallo SJ, Wang H, Prochownik EV, Lazo JS, Bahar I (2009). Discovery of novel Myc-Max heterodimer disruptors with a three-dimensional pharmacophore model. Journal of medicinal chemistry.

[49] Bentley DL, Groudine M (1986). A block to elongation is largely responsible for decreased transcription of c-myc in differentiated HL60 cells. Nature.

[50] Bacon TA, Wickstrom E (1991). Daily addition of an anti-c-myc DNA oligomer induces granulocytic differentiation of human promyelocytic leukemia HL-60 cells in both serum-containing and serum-free media. Oncogene research.

[51] Holt JT, Redner RL, Nienhuis AW (1988). An oligomer complementary to c-myc mRNA inhibits proliferation of HL-60 promyelocytic cells and induces differentiation. Molecular and cellular biology.

[52] Prochownik EV, Kukowska J, Rodgers C (1988). c-myc antisense transcripts accelerate differentiation and inhibit G1 progression in murine erythroleukemia cells. Molecular and cellular biology.

[53] Ball RK, Ziemiecki A, Schonenberger CA, Reichmann E, Redmond SM, Groner B (1988). v-myc alters the response of a cloned mouse mammary epithelial cell line to lactogenic hormones. Molecular endocrinology.

[54] Freytag SO, Geddes TJ (1992). Reciprocal regulation of adipogenesis by Myc and C/EBP alpha. Science.

[55] Onclercq R, Lavenu A, Cremisi C (1989). Pleiotropic derepression of developmentally regulated cellular and viral genes by c-myc protooncogene products in undifferentiated embryonal carcinoma cells. Nucleic acids research.

[56] Pfeiffer MJ, Esteves TC, Balbach ST, Arauzo-Bravo MJ, Stehling M, Jauch A, Houghton FD, Schwarzer C, Boiani M (2013). Reprogramming of two somatic nuclei in the same ooplasm leads to pluripotent embryonic stem cells. Stem cells.

[57] Drexler HG (1987). Classification of acute myeloid leukemias--a comparison of FAB and immunophenotyping. Leukemia.

[58] Rovera G, Santoli D, Damsky C (1979). Human promyelocytic leukemia cells in culture differentiate into macrophage-like cells when treated with a phorbol diester. Proceedings of the National Academy of Sciences of the United States of America.

[59] Fibach E, Peled T, Treves A, Kornberg A, Rachmilewitz EA (1982). Modulation of the maturation of human leukemic promyelocytes (HL-60) to granulocytes or macrophages. Leukemia research.

[60] Huang MJ, Cheng YC, Liu CR, Lin S, Liu HE (2006). A small-molecule c-Myc inhibitor, 10058-F4, induces cell-cycle arrest, apoptosis, and myeloid differentiation of human acute myeloid leukemia. Experimental hematology.

[61] Edmunds LR, Sharma L, Kang A, Lu J, Vockley J, Basu S, Uppala R, Goetzman ES, Beck ME, Scott D, Prochownik EV (2014). c-Myc programs fatty acid metabolism and dictates acetyl-CoA abundance and fate. The Journal of biological chemistry.

[62] Osthus RC, Shim H, Kim S, Li Q, Reddy R, Mukherjee M, Xu Y, Wonsey D, Lee LA, Dang CV (2000). Deregulation of glucose transporter 1 and glycolytic gene expression by c-Myc. The Journal of biological chemistry.

[63] Ward PS, Thompson CB (2012). Metabolic reprogramming: a cancer hallmark even warburg did not anticipate. Cancer cell.

[64] Zirath H, Frenzel A, Oliynyk G, Segerstrom L, Westermark UK, Larsson K, Munksgaard Persson M, Hultenby K, Lehtio J, Einvik C, Pahlman S, Kogner P, Jakobsson PJ, Henriksson MA (2013). MYC inhibition induces metabolic changes leading to accumulation of lipid droplets in tumor cells. Proceedings of the National Academy of Sciences of the United States of America.

[65] Ozawa T (1997). Genetic and functional changes in mitochondria associated with aging. Physiological reviews.

[66] Sid B, Verrax J, Calderon PB (2013). Role of AMPK activation in oxidative cell damage: Implications for alcohol-induced liver disease. Biochemical pharmacology.

[67] Gomez-Curet I, Perkins RS, Bennett R, Feidler KL, Dunn SP, Krueger LJ (2006). c-Myc inhibition negatively impacts lymphoma growth. Journal of pediatric surgery.

[68] Sampson VB, Rong NH, Han J, Yang Q, Aris V, Soteropoulos P, Petrelli NJ, Dunn SP, Krueger LJ (2007). MicroRNA let-7a down-regulates MYC and reverts MYC-induced growth in Burkitt lymphoma cells. Cancer research.

[69] Brunmair B, Staniek K, Gras F, Scharf N, Althaym A, Clara R, Roden M, Gnaiger E, Nohl H, Waldhausl W, Furnsinn C (2004). Thiazolidinediones, like metformin, inhibit respiratory complex I: a common mechanism contributing to their antidiabetic actions?. Diabetes.

[70] Owen MR, Doran E, Halestrap AP (2000). Evidence that metformin exerts its anti-diabetic effects through inhibition of complex 1 of the mitochondrial respiratory chain. The Biochemical journal.

[71] Detaille D, Guigas B, Leverve X, Wiernsperger N, Devos P (2002). Obligatory role of membrane events in the regulatory effect of metformin on the respiratory chain function. Biochemical pharmacology.

[72] Gailani D, Cadwell FJ, O'Donnell PS, Hromas RA, Macfarlane DE (1989). Absence of phorbol ester-induced down-regulation of myc protein in the phorbol ester-tolerant mutant of HL-60 promyelocytes. Cancer research.

[73] Larsson LG, Pettersson M, Oberg F, Nilsson K, Luscher B (1994). Expression of mad, mxi1, max and c-myc during induced differentiation of hematopoietic cells: opposite regulation of mad and c-myc. Oncogene.

[74] Smith MR, al-Katib A, Mohammad R, Silverman A, Szabo P, Khilnani S, Kohler W, Nath R, Mutchnick MG (1993). Prothymosin alpha gene expression correlates with proliferation, not differentiation, of HL-60 cells. Blood.

[75] Berkvens TM, Schoute F, van Ormondt H, Khan PM, van der Eb AJ (1987). Adenosine deaminase mRNA expression is regulated posttranscriptionally during differentiation of HL-60 cells. Nucleic acids research.

[76] Fujimoto T, Parton RG (2011). Not just fat: the structure and function of the lipid droplet. Cold Spring Harbor perspectives in biology.

[77] Thiele C, Spandl J (2008). Cell biology of lipid droplets. Current opinion in cell biology.

[78] Thiam AR, Farese RV, Walther TC (2013). The biophysics and cell biology of lipid droplets. Nature reviews Molecular cell biology.

[79] Mateyak MK, Obaya AJ, Sedivy JM (1999). c-Myc regulates cyclin D-Cdk4 and -Cdk6 activity but affects cell cycle progression at multiple independent points. Molecular and cellular biology.

[80] Obaya AJ, Kotenko I, Cole MD, Sedivy JM (2002). The proto-oncogene c-myc acts through the cyclin-dependent kinase (Cdk) inhibitor p27(Kip1) to facilitate the activation of Cdk4/6 and early G(1) phase progression. The Journal of biological chemistry.

[81] Wanzel M, Kleine-Kohlbrecher D, Herold S, Hock A, Berns K, Park J, Hemmings B, Eilers M (2005). Akt and 14-3-3eta regulate Miz1 to control cell-cycle arrest after DNA damage. Nature cell biology.

[82] Zajac-Kaye M (2001). Myc oncogene: a key component in cell cycle regulation and its implication for lung cancer. Lung cancer.

[83] Yin XY, Grove L, Datta NS, Long MW, Prochownik EV (1999). C-myc overexpression and p53 loss cooperate to promote genomic instability. Oncogene.

