# Supplementary material for: Structurally diverse c-Myc inhibitors share a common mechanism of action involving ATP depletion
Source: Oncotarget. 2015 May 30;6(18):15857–70. doi: 10.18632/oncotarget.4327 (PMC4599242; doi:10.18632/oncotarget.4327)
Supplement: Supplementary file 1 [file oncotarget-06-15857-s001.pdf]

Structurally diverse c-Myc inhibitors share a common mechanism of action involving ATP depletion

Supplementary Material

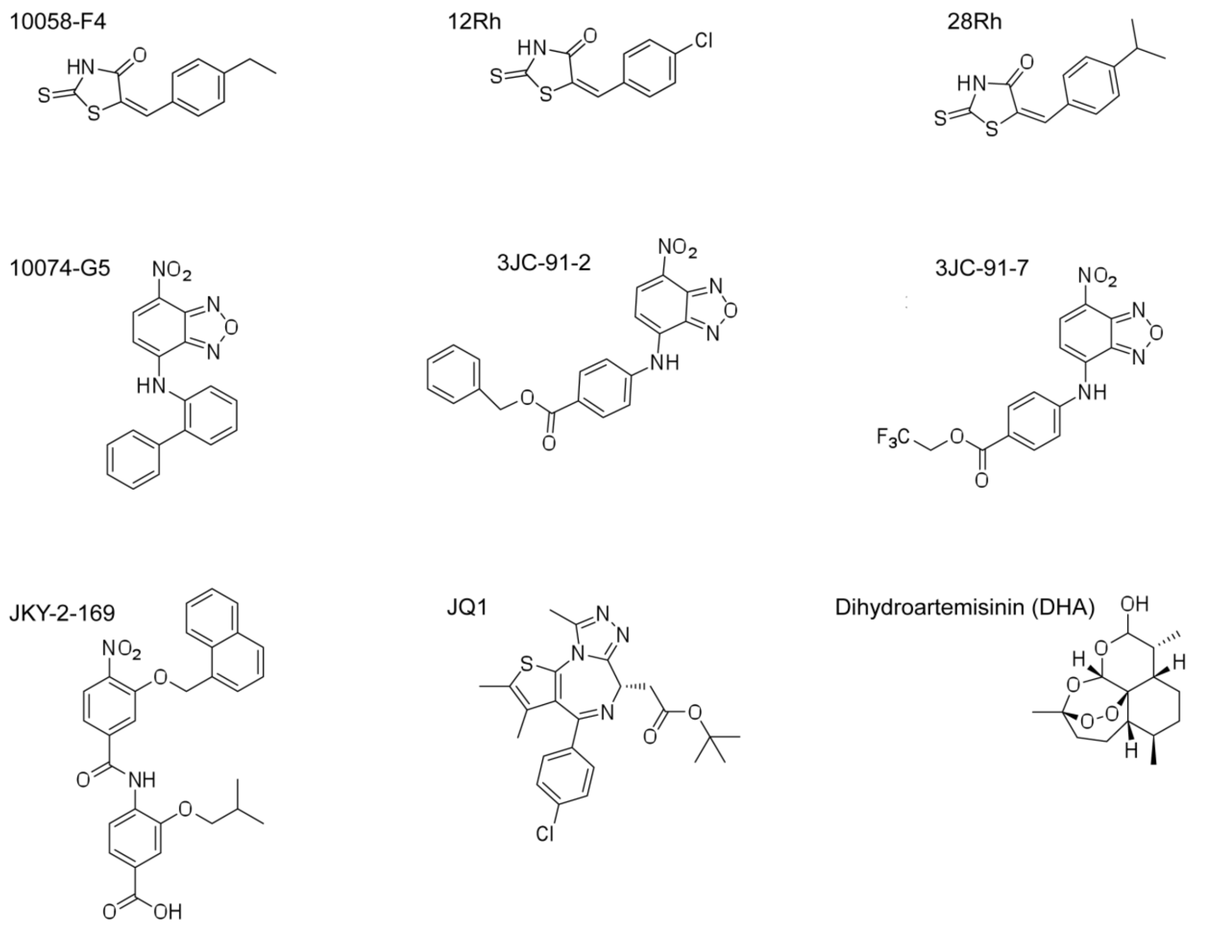

Supplementary Figure 1: Structures of 10058-F4, 12Rh, 28Rh, 10074-G5, 3JC-91-2, 3JC-91-7, JQ1, JKY-2-169 and DHA.

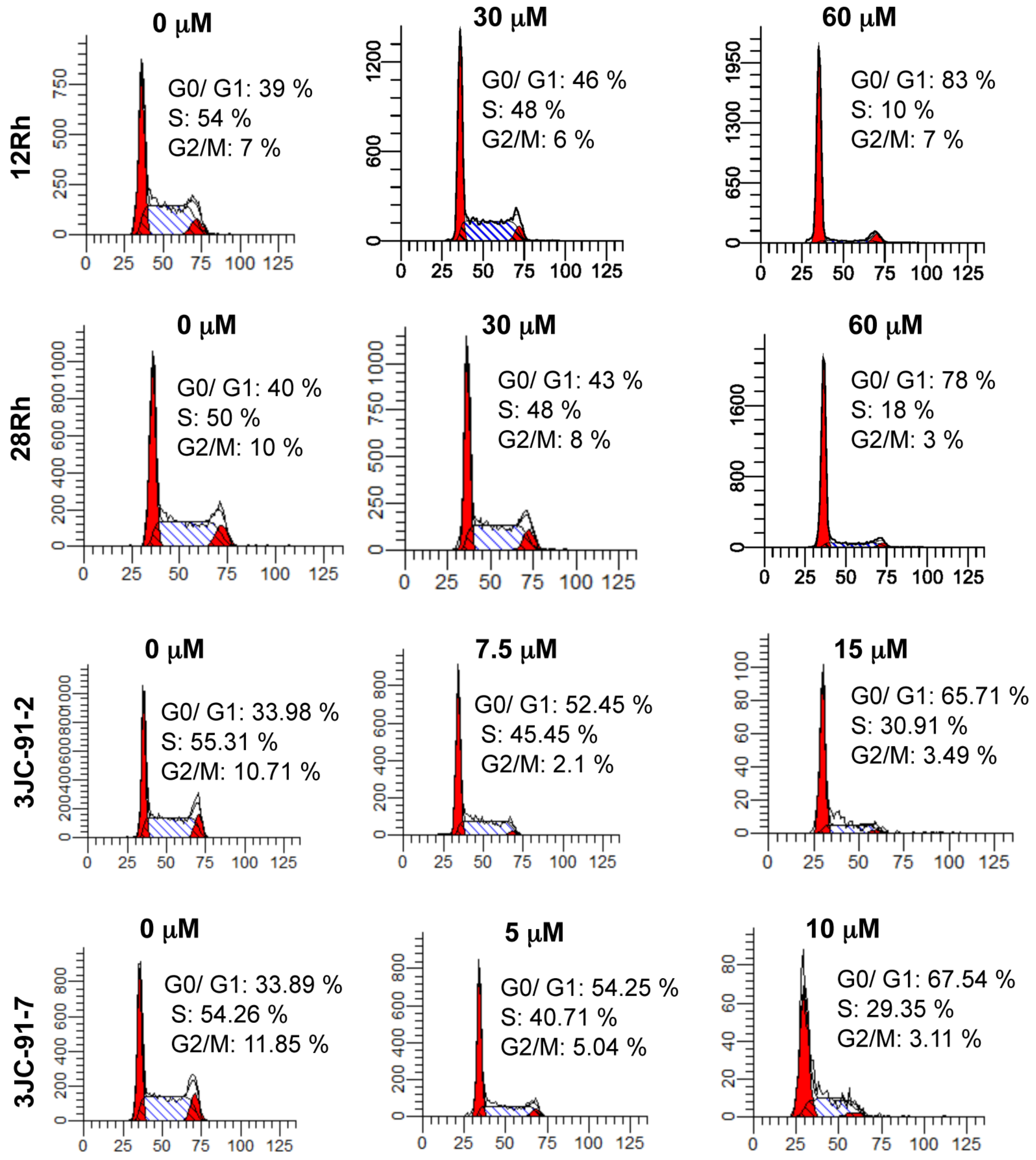

Supplementary Figure 2: Promotion of Go/G1 arrest in HL60 cells by other Myc inhibitors. HL60 cells in log-phase growth were plated into fresh medium at a concentration of ca. 10^5 cells/ml and incubated for 24-48 hr in the presence of the indicated concentrations of each inhibitor. The cells were then stained with propidium iodide and subjected to cell cycle analysis as described in Figure 1.

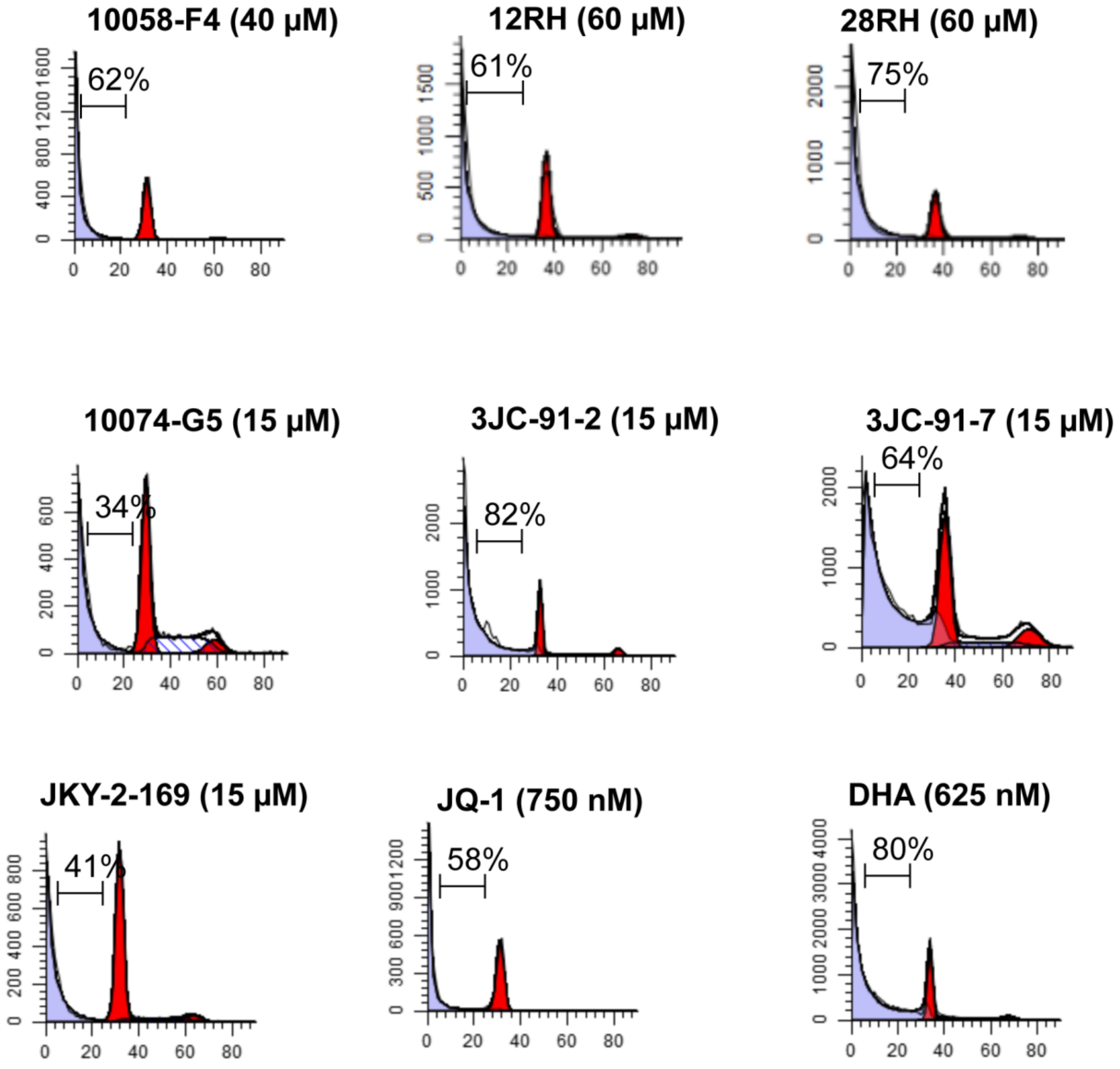

Supplementary Figure 3: Myc inhibitors eventually lead to apoptosis. HL60 cells were exposed to the indicated concentrations of Myc inhibitors for 48-72 hr, and then stained with propidium iodide as described in Figure 1 and Supplementary Figure 2. The fraction of cells containing a sub-Go/G1 DNA content is indicated.

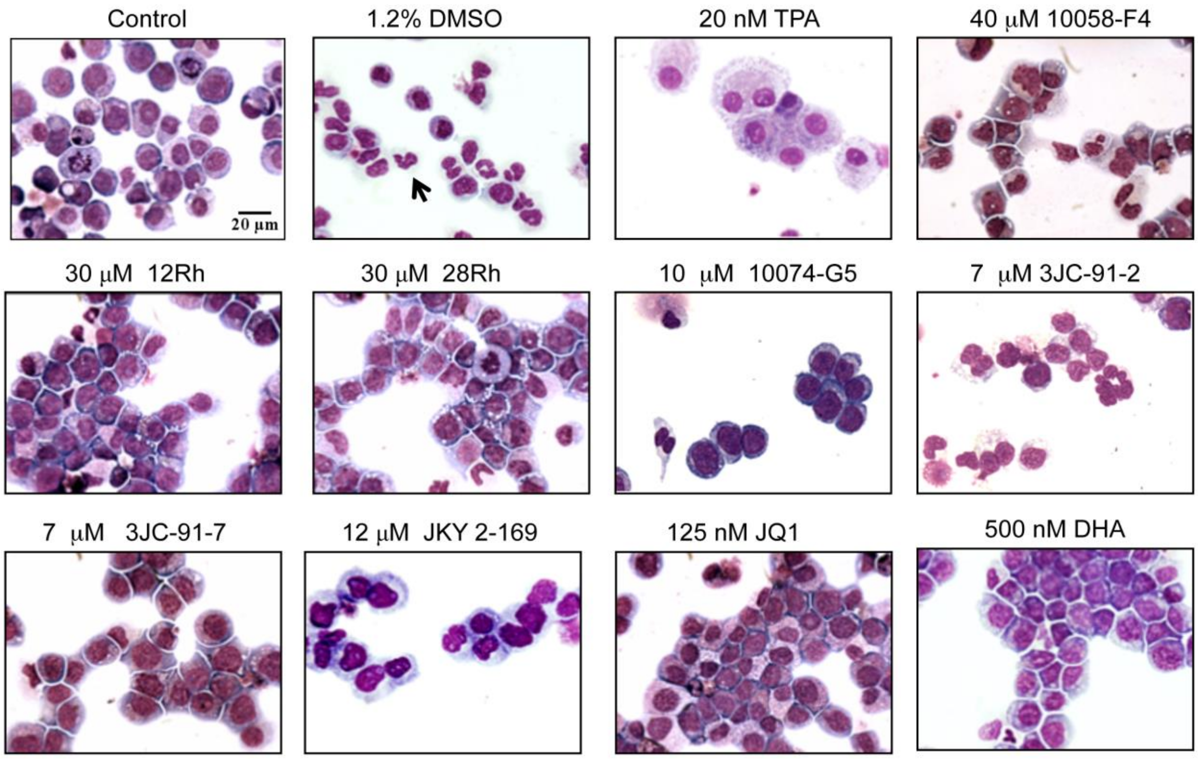

Supplementary Figure 4: Wright-Giemsa stained HL60 cells. HL60 cells were treated with the indicated concentrations of Myc inhibitors for 5 days as described in Figure 2. At the end of this time, they were pelleted by centrifugation, washed twice with PBS and then deposited on glass microscope slides using a Cyto-spin device. The cells were air-dried and stained according to standard hematologic procedures with Wright-Giemsa stain. The arrow in the panel in row 1 column 2 points to a typical mature neutrophil that is typically seen following DMSO exposure.

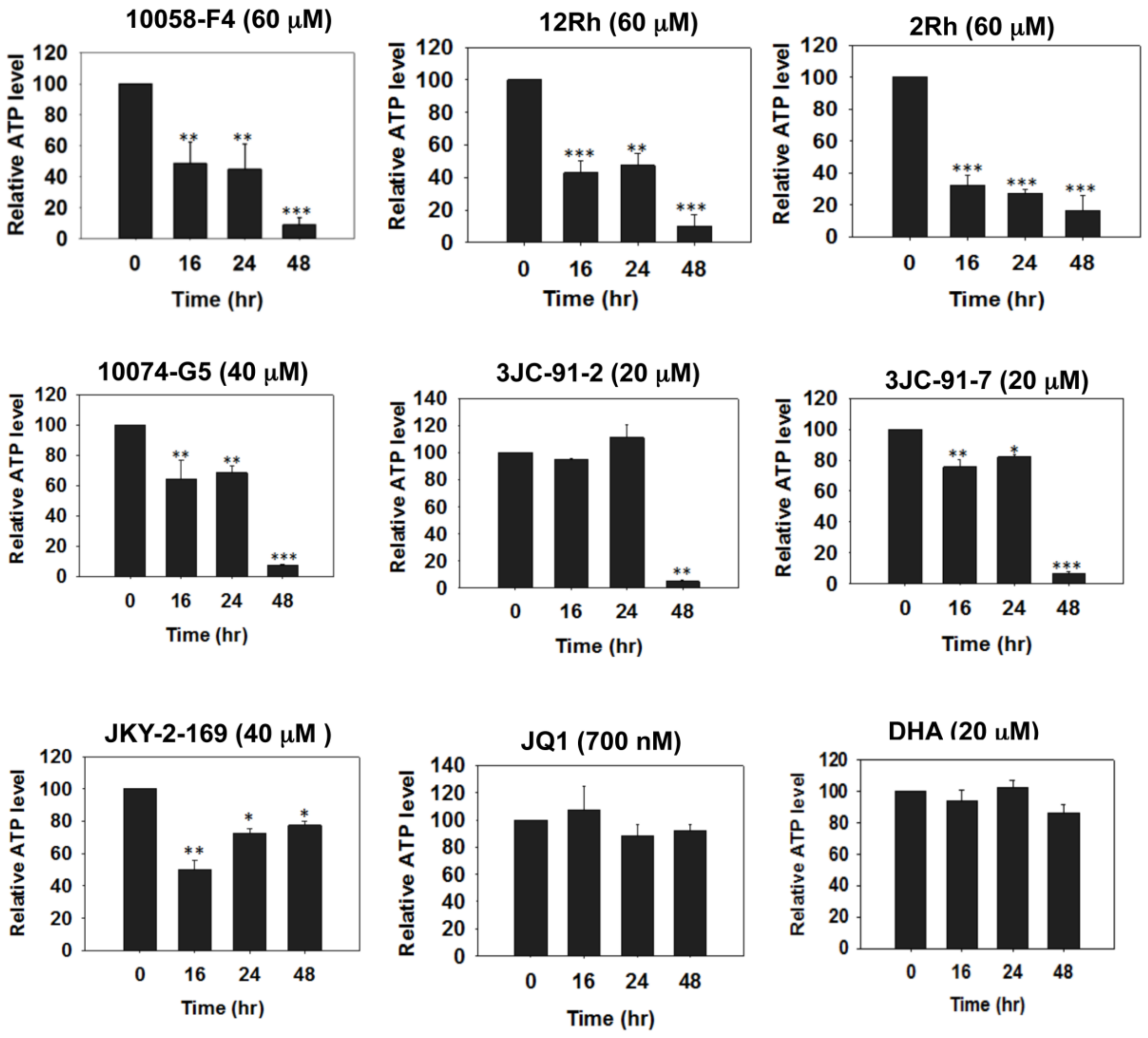

Supplementary Figure 5: Myc inhibitors promote a time-dependent loss of ATP in H460 lung cancer cells. Cells were plated in 96 well plates and exposed the following day to the indicated concentrations of Myc inhibitors for 0-48 hr. Cells were then assayed for ATP in quadruplicate as described for Figure 4. All values were adjusted to account for any differences in cell number among samples. *: $p<0.05$; **: $p<0.01$: *** : $p<0.005$.

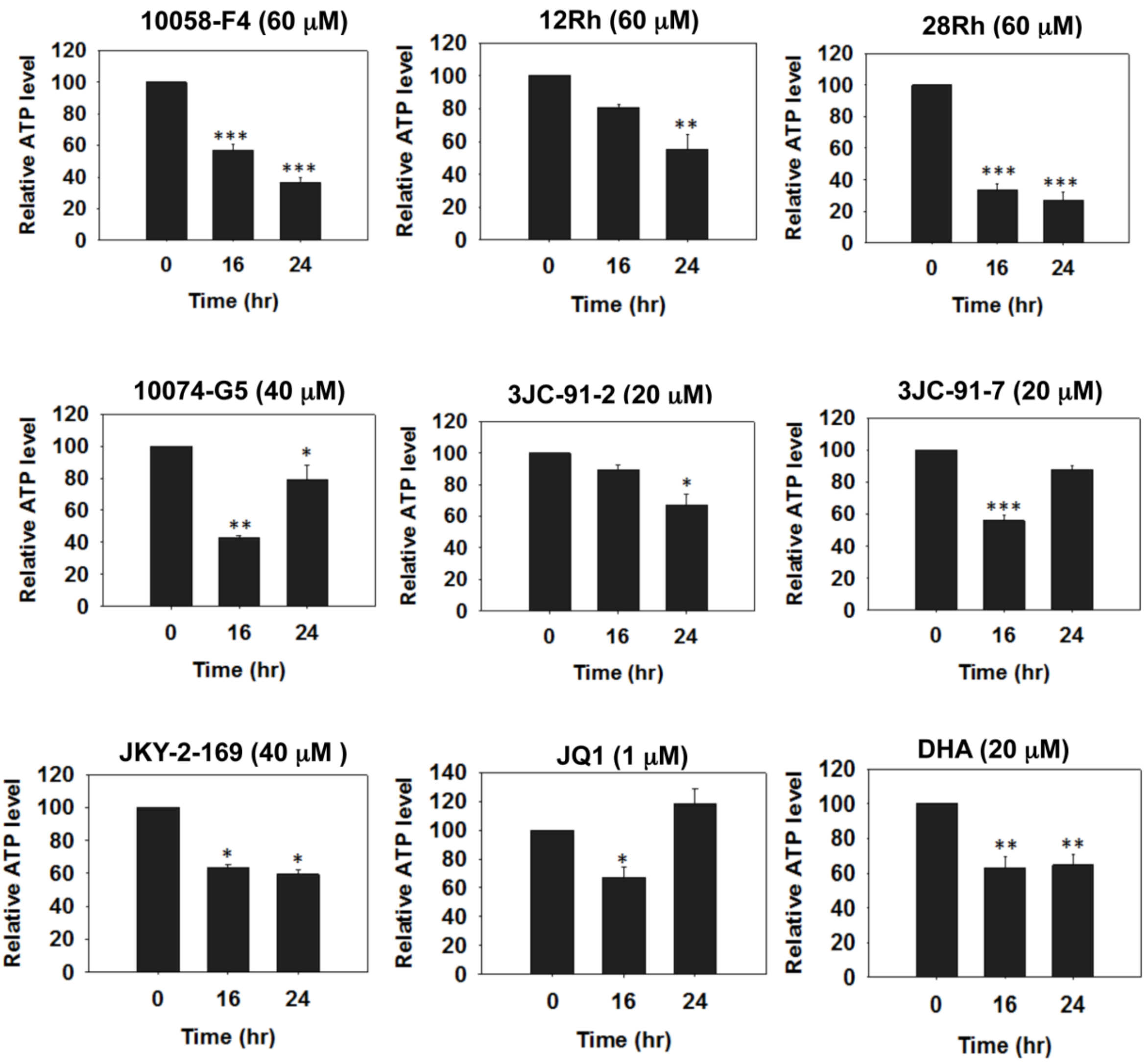

Supplementary Figure 6: Myc inhibitors promote a time-dependent loss of ATP in CaLu1 lung cancer cells. ATP assays were performed as described in the legend to Supplementary Figure 5.

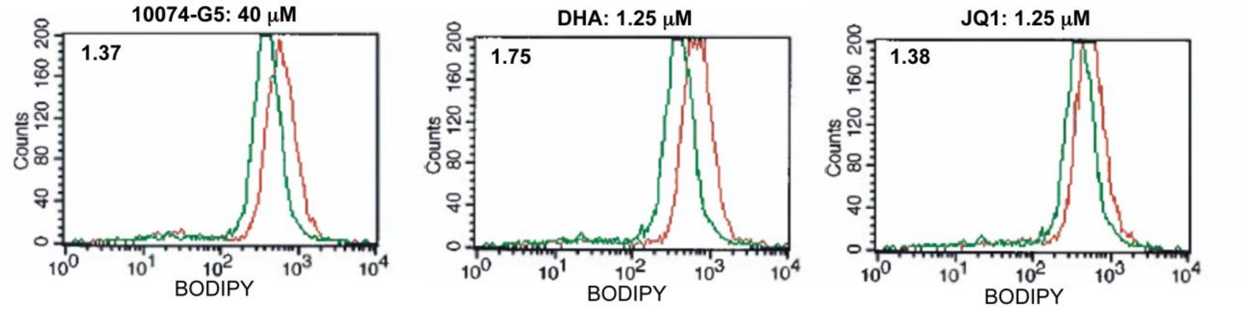

Supplementary Figure 7: JQ1 and DHA promote the accumulation of neutral lipids in HL60 cells. Logarithmically growing HL60 cells were exposed to the indicated concentrations of Myc inhibitors for 2 days at which time they were stained with BODIPY-493/503 as described for H460 cells (Figure 3). Cells were also exposed to 10074-G5 as a positive control.

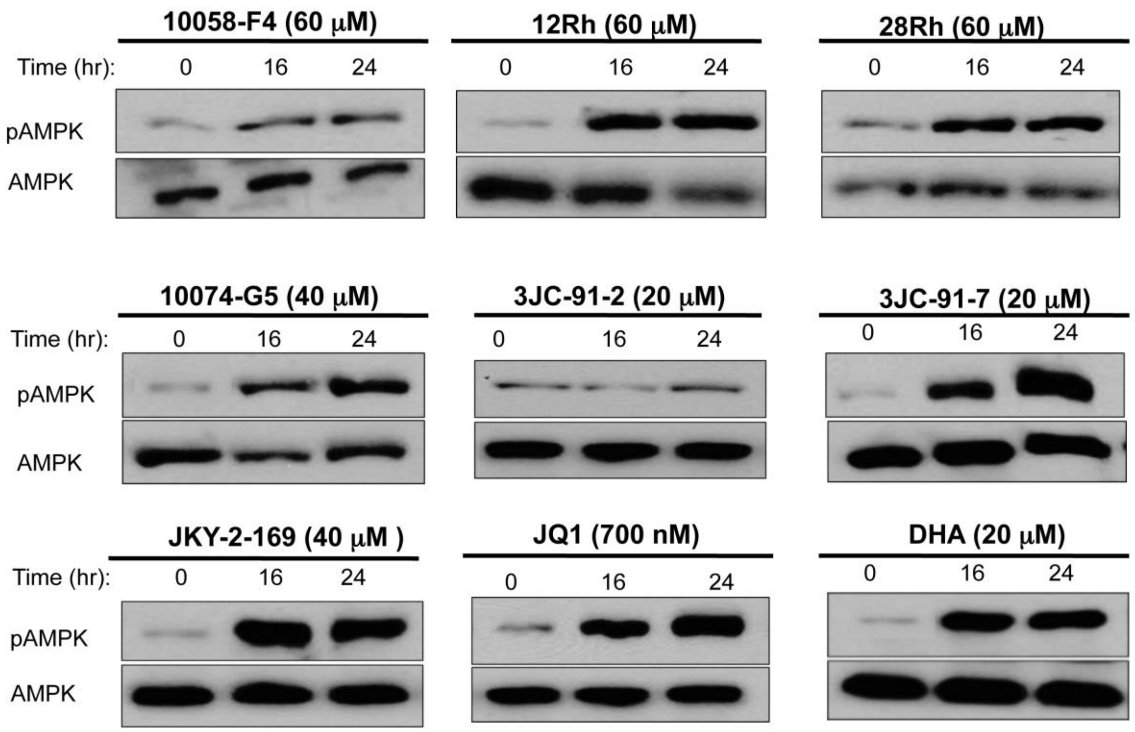

Supplementary Figure 8: Myc inhibitors activate AMPK in H460 lung cancer cells. Myc inhibitors were added to log-phase cells for the indicated periods of time. The cells were then harvested and immuno-blotted for total AMPK or pAMPK as described for Figure 5.

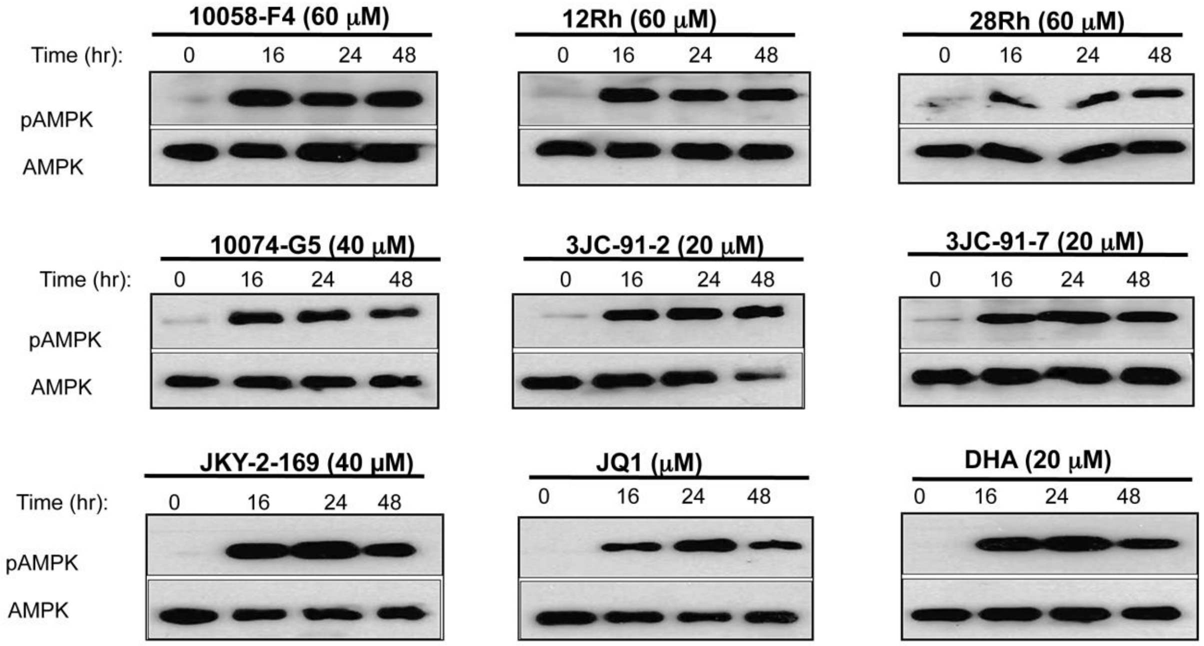

Supplementary Figure 9: Myc inhibitors activate AMPK inCaLu1 lung cancer cells. Myc inhibitors were added to log-phase cells for the indicated periods of time. The cells were then harvested and immuno-blotted for total AMPK or pAMPK as described for Figure 5.

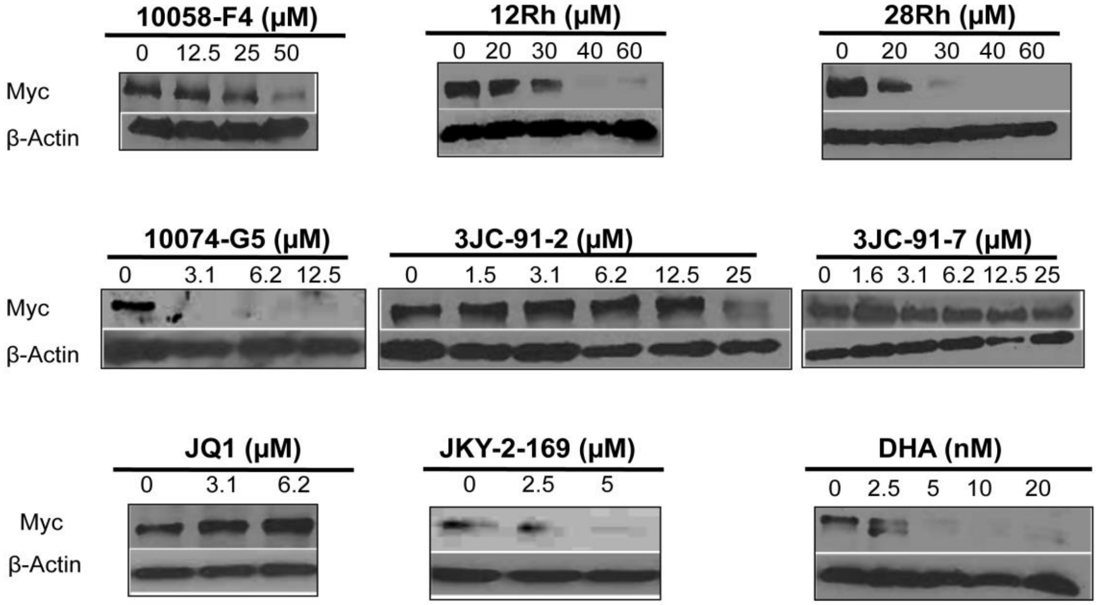

Supplementary Figure 10: Myc protein immunoblots in H460 lung cancer cells. Cells were exposed for 48 hr to the indicated concentrations of Myc inhibitors. Cells were then assessed for Myc protein by standard immuno-blotting.

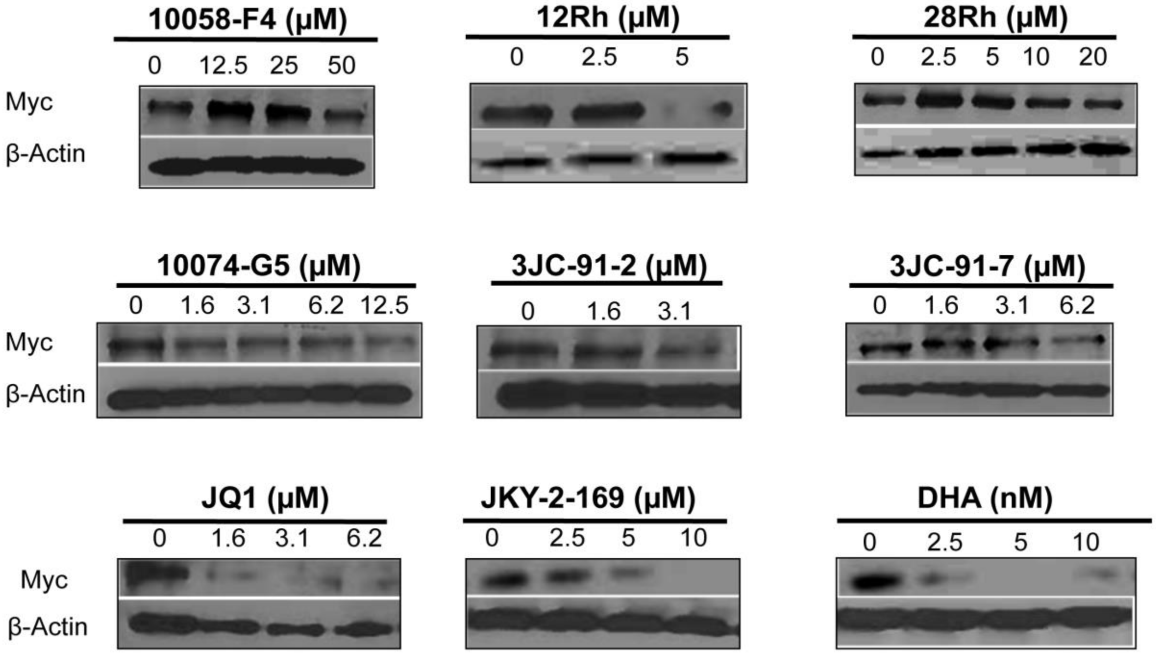

Supplementary Figure 11: Myc protein immunoblots in CaLu1 lung cancer cells. Cells were exposed for 48 hr to the indicated concentrations of Myc inhibitors. Cells were then assessed for Myc protein by standard immuno-blotting.
